# Zero-shot prompt-based video encoder for surgical gesture recognition

**DOI:** 10.1007/s11548-024-03257-1

**Published:** 2024-09-17

**Authors:** Mingxing Rao, Yinhong Qin, Soheil Kolouri, Jie Ying Wu, Daniel Moyer

**Affiliations:** Department of Computer Science, Vanderbilt University, Nashville, USA

**Keywords:** Surgical gesture recognition, Prompt engineering, Zero-shot learning, Cross-task learning

## Abstract

**Purpose:**

In order to produce a surgical gesture recognition system that can support a wide variety of procedures, either a very large annotated dataset must be acquired, or fitted models must generalize to new labels (so-called zero-shot capability). In this paper we investigate the feasibility of latter option.

**Methods:**

Leveraging the bridge-prompt framework, we prompt-tune a pre-trained vision-text model (CLIP) for gesture recognition in surgical videos. This can utilize extensive outside video data such as text, but also make use of label meta-data and weakly supervised contrastive losses.

**Results:**

Our experiments show that prompt-based video encoder outperforms standard encoders in surgical gesture recognition tasks. Notably, it displays strong performance in zero-shot scenarios, where gestures/tasks that were not provided during the encoder training phase are included in the prediction phase. Additionally, we measure the benefit of inclusion text descriptions in the feature extractor training schema.

**Conclusion:**

Bridge-prompt and similar pre-trained + prompt-tuned video encoder models present significant visual representation for surgical robotics, especially in gesture recognition tasks. Given the diverse range of surgical tasks (gestures), the ability of these models to zero-shot transfer without the need for any task (gesture) specific retraining makes them invaluable.

## Introduction

Proposed intra-operative robotic gesture recognition systems are described as enabling automation, rapid skill assessment and pedagogic feedback, and more general surgical support [1]. However, current methods [2–4] for gesture recognition attempt to estimate a fixed set of supervised gestures from contemporaneously collected video and/or kinematic datastreams [5–8]. These systems are often trained in a fully supervised manner, which requires frame-wise gesture labels for the entire training set, and importantly require specification of the target labels during the whole training phase.

We believe that in order to produce a system that can provide support to wide variety of procedures, gesture recognition as a sub-field will either need (a) a large number of annotated datasets for each procedure as previous fully supervised methods demand, or (b) a system that can generalize well to new label sets with limited supervision. The latter option is likely more efficient given the expense of annotation and fits within the “zero-shot learning” paradigm, where a general representation is reused for labelling tasks unseen during training [9].

We thus investigate the feasibility of such a zero-shot gesture recognition method using weak supervision and text augmentation. We focus on improving visual feature extraction from video data streams, as these data offer rich information about surgical gestures (surgemes) and kinematic data require access to the research API, limiting current use cases. We use the bridge-prompt [10] framework which specifically relaxes the fully supervised constraints: Bridge-Prompt is able to use large weakly supervised datasets, which are relatively inexpensive and numerous in comparison with densely annotated data, and our experiments suggest that Bridge-Prompt generalizes well to unseen labels (i.e. novel gestures).

In order to validate these claims and evaluate Bridge-Prompt’s overall efficacy and zero-shot capability, we demonstrate usage on the JIGSAWS [11] and RARP-45 [6] dataset, simulating both standard and zero-shot use cases on cross-validated training schemes. We compare image encoders with differing configurations, varying experimentally the amount of label information (both annotation presence/absence and label text). We then compare the performance of experimental cases and standard baselines from the literature using a standard prediction recognition model (MS-TCN++, [12]). Our experiments show the benefit of using Bridge-Prompt image encoders for gesture recognition tasks.

In summary, in the present work, we do the following:

- We demonstrate the within-task and zero-shot capability of bridge-prompt.
- We show that gesture labels’ text descriptions do not improve training.
- We provide open-source code^1^ for prompt-tuning encoders with bridge-prompt.

### Terminology

Throughout this manuscript we use the phrase “*pre-trained*” exclusively to denote previously trained instances of encoder networks that we either directly use without modifying or further train using a different weakly supervised loss function. We refer to this further training phase as “*prompt tuning*”.

No matter if an encoder is only pre-trained or incorporates prompt-training, its weights are then frozen and used unchanged for the *supervised training* phase, wherein a supervised network will be trained to predict gesture labels from the frozen embeddings.

## Related work

Surgical robotic gesture recognition has an extensive history of study [13–15]. While multiple disparate modalities are each reasonable to incorporate into a recognition system, video is by far the most popular [5, 7, 16, 17], followed by integration of video with robotic kinematic data [6, 18, 19], and discrete surgical event streams [20]. Notably, almost all proposed methods rely on video data, or on video derived features such as optical flow [21].

Gesture recognition is usually considered in a temporal context, and thus many of the prediction models are taken from time-series prediction and forecasting domains: HMM, LSTMs, temporal convolution, and attention methods [6, 22–24]. The Temporal Convolutional Network (TCN) has emerged as a common selection for the majority of deep learning-based temporal gesture studies [12, 24, 25]. MS-TCN and MS-TCN++ [12, 25] are the most relevant instances of this family of temporal modelling methods, as they were specifically designed for temporal action segmentation, and have been used in several surgical workflow analysis tasks [26, 27]. Our objective is to test the effectiveness of differing image encoders and not the prediction head architectures; thus, we use a generic MS-TCN++ implementation throughout the experiments.

Feature extraction has become an essential part of deep learning systems [28], particularly in computer vision [29]. Initially performed by fully convolutional architectures trained in a supervised end-to-end schema, recent image encoders are typified by extended pre-training [30] using proxy tasks and/or self-supervised, contrastive methods. In surgical video, standard methods include Inflated-3D [2], 3DCNN [3] and 3DResNet [4]; these methods fall into the category of fully convolutional supervised end-to-end trained encoders. Bridge-Prompt, which uses the pre-trained CLIP [29] vision-text joint embedding model, has been proposed as a generic video encoder. The objective of this paper is to measure both bridge-prompt’s performance on the standard surgical gesture task and its zero-shot generalization ability.

### Zero-shot learning

In traditional learning, models learn from labelled samples for each class and make predictions on previously unseen samples of the same classes. In zero-shot learning, models are trained on a subset of classes and tested on a different set of classes without any overlap [9]. This is especially crucial in surgical robot videos where collecting annotated gestures for every possible class is impractical or expensive, leading to a vast amount of surgical video being unlabelled.

## Method

This section describes the two parts of our model: (1) the video encoder (bridge-prompt) and (2) the downstream gesture recognition model. The former we construct during prompt-tuning and is the focus of our empirical study, while the latter is used in evaluation of each of the encoders, including both the proposed encoder (bridge-prompt) and baseline encoders. Though neither is first proposed by this manuscript, we include descriptions of both, as understanding their nuances (or simplicity, in the case of the downstream model) is necessary for contextualizing experiments.

### Bridge-prompt prompt-tuning architecture

**Fig. 1:**
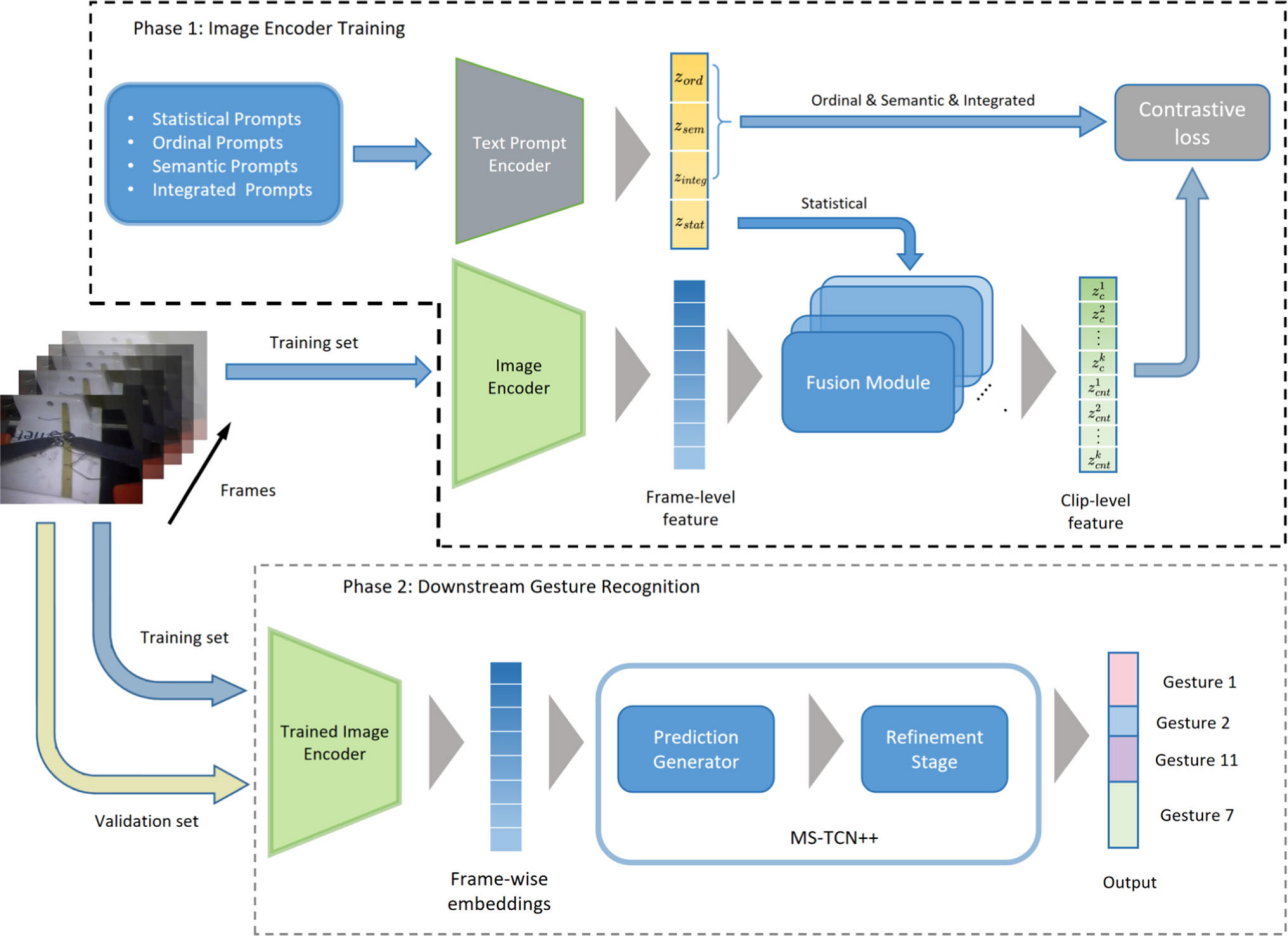
The two phases of our training schema: at top the Bridge-Prompt pre-training, at bottom a “simple probe” predictor measuring performance on the supervised gesture recognition task

Bridge-prompt is a training protocol for constructing high-quality image encoders for sequential labelling of video frames. It starts with an image-text joint encoder, for which the standard model is the CLIP model [29], which is in turn based on ViT-B/16 vision transformer trained on three large natural image datasets [31–33], alongside an analogous text transformer to GPT-2 [34]. In the CLIP training protocol, these two models were modified to have matching encodings, so that from either the image or text one could predict the other. Bridge-Prompt starts at this pre-trained CLIP state and prescribes additional video sequence-based training. This fine-tuning to surgical video (and to the particular surgical gesture labels) is the first phase of our empirical work (Fig. 1).

We follow the Bridge-Prompt protocol and first split videos into sub-videos with a fixed number of frames, but possibly at different sampling rates. All resulting sub-videos have the same number of frames, alongside a label for each frame (which may be missing/undefined). Every defined label may also have a text description, e.g. “orienting needle” or “pulling suture with left hand”, though this may also be left undefined, or, as we experiment with, replaced with a categorical placeholder.

For each sub-video four text prompts are constructed from the sub-video’s labels:

1. **Statistical text prompt**: “*this video contains K actions in total*”
2. **Ordinal text prompt**: “*this is the* *i*th *action in the video*” (defined for each distinct label interval in the video)
3. **Semantic text prompt**: “{$Ord_i$}, *the person is performing* {*Gesture* *i* *text description}*” where $Ord_i$ refers to “*Firstly*”^2^, “*Secondly*”, etc.
4. **Integrated text prompt**: the concatenation of all of the semantic text prompts.

These prompts are then sent to the text encoder, to form $z_{\textrm{stat}}$, $z_{\textrm{ord}}$, $z_{\textrm{sem}}^k$ and $z_{\textrm{int}}$, respectively, where *k* represents *k*-th gesture in a video clip.

Each image frame $x_t$ is passed through the initial image encoder *f*; this is the encoder that will be reused for the downstream surgical gesture recognition task. However, for fine-tuning only, the encodings $f(x_t)$ are then processed as a sequence by a “fusion module”, which also receives as input the ordinal text prompts ($z_{\textrm{ord}}$) and summary statistic frame-level indicators (count tokens, split indicators, and length/position indicators). The outputs of the fusion module (the “fusion encodings”) include $z_c^k$ for each gesture, mean-pooled $\bar{z}_c$, and a separate embedding $z_{\textrm{count}}$. $z_{\textrm{count}}^k$ is output at sub-clip for each gesture, but only the mean-pooled aggregate is used. These encodings are the focus of the loss components which drive the contrastive fine-tuning.

### Contrastive pre-training losses

For two vectors $z_x,z_y$ on the same space the cosine similarity is

1 $$\begin{aligned} \text {s}(\mathbf {z_x},\mathbf {z_y})=\frac{\mathbf {z_x} \cdot \mathbf {z_y}}{\Vert \mathbf {z_x}\Vert \Vert \mathbf {z_y}\Vert } \end{aligned}$$

We can then define a batch similarity matrix from sets $Z_x = \{z_{x,b}\}$ and $Z_y = \{z_{y,b}\}$

2 $$\begin{aligned} \text {S}(Z_x, Z_y) = \begin{bmatrix} \text {s}(z_{x1}, z_{y1}) & \quad \dots & \quad \text {s}(z_{x1}, z_{yB}) \\ \vdots &  \quad \ddots & \quad \vdots \\ \text {s}(z_{xB}, z_{y1}) & \quad \dots &  \quad \text {s}(z_{xB}, z_{yB}) \end{bmatrix} \end{aligned}$$

where *b* denotes the sub-video (batch) index and *B* is the batch size. We construct three different similarity matrices:

$S_{\textrm{sem}}^k =\text {S}(Z_{c}^k,Z_{\textrm{sem}}^k)$ the similarity between the frame-wise encodings and the semantic text prompt embeddings, $S_{\textrm{int}} = \text {S}(\bar{Z}_c,Z_{\textrm{int}})$ the similarity between the mean-pooled embeddings and the integrated text prompt embeddings, and $S_{\textrm{stat}} = \text {S}(\bar{Z}_{\textrm{count}},Z_{\textrm{stat}})$ the similarity between the mean-pooled count embeddings and the statistical text prompt embeddings. After computing each similarity matrix soft-max is applied first row-wise/column-wise to form text-wise/clip-wise $\bar{S}_{\textrm{sem}},\bar{S}_{\textrm{int}}$, and $\bar{S}_{\textrm{stat}}$, respectively.

Within each batch, matching image-text pairs are taken as positive contrastive pairs, while mismatched pairs (i.e. videos paired with label text from a different video) are taken as negative contrastive pairs; this is to say that in the context of our contrastive learning problem we optimize the matrices towards the identity matrix. Towards this end we define three losses for each of the three matrices:

3 $$\begin{aligned} \mathcal {L}_{\textrm{sem}}^k&= \frac{1}{2}[{D}[ S_{\textrm{sem}}^k \Vert I] + {D}[ I \Vert S_{\textrm{sem}}^k]] \nonumber \\ \mathcal {L}_{\textrm{stat}}&= \frac{1}{2}[{D}[ S_{\textrm{stat}} \Vert I] + {D}[ I \Vert S_{\textrm{stat}}]] \\ \mathcal {L}_{\textrm{int}}&= \frac{1}{2}[{D}[ S_{\textrm{int}} \Vert I] + {D}[ I \Vert S_{\textrm{int}}]]\nonumber \end{aligned}$$

where the (generalized KL) divergence *D* is defined for square matrices of matching dimension

4 $$\begin{aligned} {D}[A \Vert B] = \sum _{i=1}^N \sum _{j=1}^N A_{ij} \log \frac{A_{ij}}{B_{ij}}. \end{aligned}$$

### Gesture recognition model

To measure the effectiveness of prompt-based video encoder, during the evaluation we freeze the weights in the video encoder and do frame-wise visual embedding. The frame encoder is fixed, and we train a predictive model for gesture recognition based on that fixed visual embedding. Our chosen downstream predictive model is the MS-TCN++ [12] temporal convolution networks have become a common choice in action segmentation, and the MS-TCN++ is a refinement of the original MS-TCN. MS-TCN++ uses a simple two-stage training and a slightly modified convolutional configuration with dilations. We avoid deeper or more nuanced architectures (such as those incorporating attention, or longer context windows) to ensure that performance is due to the quality of the features and not the complexity of the classifier itself.

**Table 1:** Here we present gesture recognition results on the JIGSAWS dataset for 3DResNet, I3D, and bridge prompt (BP) with both ResNet50 and ViT variants, as well as the bridge prompt architecture with no JIGSAWS-specific prompt-tuning (CLIP)

	Knot Tying			Suturing		
	Acc	Edit	F1@{10, 25, 50}	Acc	Edit	F1@{10, 25, 50}
3DResNet	66.3	65.3	69.7, 65.56, 51.14	68.19	67.78	73.37, 69.06, 57.87
I3D	68.39	74.36	78.20, 70.27, 54.21	68.63	75.93	78.44, 74.28, 59.69
CLIP-ViT	70.42	75.52	77.41, 72.52, 59.77	75.15	78.81	83.35, 79.94, 68.02
CLIP-ResNet50	69.05	76.54	79.06, 73.21, 59.55	71.04	77.91	81.35, 77.28, 64.57
BP-ResNet50	77.82	**78**.**97**	**84**.**79**, **81**.**03**, 66.39	81.42	**84**.**71**	**89**.**12**, **87**.**71**, 77.60
BP-ViT	**81**.**00**	74.19	81.19, 78.58, **68**.**31**	**81**.**72**	83.89	87.34, 85.57, **77**.**61**
MA-TCN[C]	Causal variant from [6], see footnote 4.			83.4	81.6	87.7 only F1@10 given

Best performance referring to specific metric and task

We provide the MA-TCN[C] numbers reported by van Amsterdam et al. [6] for overall context, but MA-TCN[C] uses both the video and kinematic data streams and a more complex prediction model

## Experiments

### Datasets, implementation, training and evaluation

*Datasets* We demonstrate Bridge-Prompt and baseline methods on two standard datasets: the JHU-ISI gesture and skill assessment working set (JIGSAWS) [11], which is composed of endoscopic video of suturing and knot-tying in a phantom environment, and robot-assisted radical prostatectomies (RARP-45) [6], which is composed of endoscopic video recordings of prostatectomies. Both datasets are collected from 8 surgeons with varying skill levels using the da Vinci Surgical System (dVSS, Intuitive Surgical, Sunnyvale, CA, USA) and are annotated for multiple gestures at the image-frame level: 15 gestures at 30 Hz for JIGSAWS and 8 gestures at 60 Hz for RARP-45. JIGSAWS additionally has robotic kinematic recordings, but we do not use these data in Bridge-Prompt trained methods. We also omit the JIGSAWS needle passing task due to data quality.

**Table 2:** Here we present gesture recognition results on the RARP-45 dataset for 3DResNet, I3D, and bridge prompt (BP) with the ViT variant, as well as a “no RARP-45-specific prompt-tuning” case (CLIP). BP-ResNet50 failed to converge; thus, we decide not to post it

	Acc	Edit	F1@10	F1@25	F1@50
3DResNet	66.97	**76**.**95**	71.76	66.34	52.90
I3D	65.95	74.41	71.52	65.35	51.23
CLIP-ViT	70.00	73.88	74.94	73.30	59.95
CLIP-ResNet50	68.93	71.85	70.83	66.90	55.09
BP-ViT	**77**.**36**	72.16	**76**.**29**	**74**.**50**	**65**.**32**

Best performance referring to specific metric and task

*Implementation* We implement multiple Bridge-Prompt variants as well as two baseline image encoders (3DResNet [4] and I3D [2]) in PyTorch. Each Bridge-Prompt implementation is composed of a backbone image encoder (either the default ViT-B/16 [35] or ResNet-50 [36]) and the analogous GPT-2 text encoder [34] which is discarded after training. These backbones are initialized using standard pre-trained weight-sets.^3^ The Bridge-Prompt encoders are then prompt-tuned using the Adam optimizer minimizing the sum of the losses in Eq. 3 in the prompt-tuning phase.

After prompt-tuning we freeze the weights of each image encoder and then train a standard prediction network for the supervised task (MS-TCN++ [12]). This second directly supervised phase we call the *supervised training phase*. The supervised training phase has its own hold-out test set, and it is on this hold-out that we report performance metrics. For JIGSAWS we use leave-one-user-out (LOUO) [11] cross-validation, and for RARP-45 we choose 10 videos (out of 36 total) as the test set.

*Training samples* Even though we are constructing frame-by-frame image encoder, their training requires multi-frame segments (video clips) and their corresponding sequence of gesture labels. Each video clip is sampled from the original video of JIGSAWS or RARP-45 in 16 frame windows at three separate temporal sampling rates (sampled frames every 4/8/16 frames for JIGSAWS and 6/15/30 frames for RARP-45). We resize each frame to $224\times 224$ pixels. The input format for the video clips for all methods (Bridge-Prompt [10], I3D [2], 3DResNet [4]) is the same, and input sets are changed only by the stated experimental condition. For the label sequences from JIGSAWS, we provide two additional placeholder labels/prompts for the unlabelled frames at the beginning and end of each video: “Waiting and preparing for the surgery” for beginning frames and “Finishing the surgery” for ending frames.

*Training time* All experiments were run on NVIDIA A40 GPUs or NVIDIA A5000 GPUs. The prompt-tuning phase for all Bridge-Prompt variants was conducted on two GPUs; otherwise, only a single GPU was used. In JIGSAWS, pre-training for 50 epochs for each tested variant of Bridge-Prompt takes approximately 8 h using two A40 GPUs. It takes 5 min to train the MS-TCN++ during the supervised training phase, using pre-extracted image encodings.

*Performance metrics* We assess the outcomes using five standard evaluation metrics, Accuracy (Acc.), Edit Distance, and F1@{10, 25, 50} [24]. For JIGSAWS we condition this on pre-training task (Knot Tying or Suturing), and for zero-shot cases we also condition on each unseen label.

**Table 3:** Above the double line, we show zero-shot gesture recognition on JIGSAWS with different sets of pre-trained labels (%), along with an All gestures supervised case for reference, and encoders using indices (e.g. “Gesture 2”) instead of text descriptions during prompt-tuning (“ind”). “No gestures” has no prompt-tuning

	Knot tying			Suturing		
	Acc	Edit	F1@{10, 25, 50}	Acc	Edit	F1@{10, 25, 50}
No gestures	70.42	75.52	77.41, 72.52, 59.77	75.15	78.81	83.35, 79.94, 68.02
5 gestures	79.60	73.90	81.34, 78.54, 63.15	81.00	82.80	87.31, 85.74, 76.25
5 gestures (ind)	78.93	76.10	82.63, 78.82, 66.30	79.63	81.55	85.10, 83.14, 72.50
10 gestures	77.07	76.56	81.79, 77.54, 66.23	81.34	82.45	87.76, 85.77, 77.86
10 gestures (ind)	77.49	75.19	80.82, 77.05, 65.27	81.91	82.42	87.92, 85.08, 77.10
All gestures	81.00	74.19	81.19, 78.58, 68.31	81.72	83.89	87.34, 85.57, 77.61
Cross-task	76.60	68.90	77.60, 74.16, 60.80	78.84	81.85	85.63, 83.58, 73.77

Below the double line, we show cross-task zero-shot generalization, using the Knot-Tying prompt-tuned model for Suturing supervised training/evaluation, and vice versa

### Experimental conditions

We first measure the quality of the features learned by the various encoders operating under normal (nonzero-shot) conditions. Results are reported in Tables 1 and 2. These results show that Bridge-Prompt improves gesture recognition performance as measured by all but one performance metric. Further, due to the similarity between Bridge-Prompt performance with either ResNet50 or ViT backbones, this performance gain does not appear to be due to the transformer architecture of ViT. However, ResNet50 backbones appear to be slightly less stable in RARP-45 training; across multiple runs we experienced exploding gradients, and the method failed to converge. Tuning the learning-rate might resolve this issue, but we did not have the resources to tune that parameter. We include JIGSAWS results reported in van Amsterdam et al. for contextualization,^4^ as we believe this to be a state-of-the-art contemporary system utilizing all available data streams (both visual and kinematic), and (presumably) many model architecture optimizations and procedure refinements.

*Zero-shot capability* We measure the efficacy of the Bridge-Prompt image encoder for describing unseen gestures, i.e. zero-shot generalization capability. This is done by selectively training encoders using only subsets of the gestures: for JIGSAWS, we test subsets with only gestures 1–5, only gestures 1–10, and no gestures at all (the “pure CLIP [29] encoder”), presenting results in Table 3. We disaggregate performance by task, gesture and model in Table 5. The 1–10 gesture model necessarily contains 6 and 8, and thus performance is not reported. For RARP-45 we test subsets with only gestures 1–3 and gestures 1–6, presenting results in Table 4. Per-gesture performance disaggregation is provided as a table in Appendix. For JIGSAWS, we also trained video encoders on one task but evaluated them on the other (“cross-task”) and reported at the bottom of Table 3. This experiment is not possible in RARP-45, as it only has one task. In general we find that Bridge-Prompt prompt-tuning with only a subset of gestures or even on a different task still provides improvement for most performance measures. This, to us, indicates that Bridge-Prompt has relatively high zero-shot capability.

**Table 4:** Here we show zero-shot surgical gesture recognition on RARP-45 with different sets of pre-trained labels (%) on RARP-45 dataset, and the same encoders using indices (e.g. “Gesture 2”) instead of text descriptions during prompt-tuning (“ind”)

	Acc	Edit	F1@10	F1@25	F1@50
3 gestures	77.78	75.09	80.96	79.28	70.60
3 gestures (ind)	78.93	75.80	80.82	79.38	69.78
6 gestures	79.19	71.85	76.60	73.68	65.17
6 gestures (ind)	79.73	76.51	80.70	79.06	69.01

**Table 5:** We show zero shot F1@10 scores for single gestures on JIGSAWS with different sizes of pre-training sets (%) that *exclude* those gestures, along with an All gestures supervised case for reference, and encoders using indices (e.g. “Gesture 2”) instead of text descriptions during prompt-tuning (“ind”)

	Knot tying					Suturing		
	G11	G12	G13	G14	G15	G6	G8	G11
No gestures	89.75	65.31	59.47	60.07	84.29	58.23	46.27	75.90
5 gestures	91.31	75.76	70.69	73.39	89.92	79.07	69.46	92.18
5 gestures (ind)	90.65	75.98	69.00	71.28	88.48	78.68	67.66	83.04
10 gestures	91.79	72.89	65.87	67.21	88.68	–	–	89.16
10 gestures (ind)	92.75	71.70	70.59	70.44	86.84	–	–	87.97
All gestures	93.61	76.55	72.17	73.49	91.01	81.20	68.11	92.86

*Text description ablation* Finally, we show evidence that text descriptions provide negligible benefit for bridge-prompt encodings. We measured the value of gesture text descriptions by ablating them to simple “Gesture Index” categorical descriptors. For example, gesture 9 could be described as “using right hand to help tighten suture” or as “Gesture 9”. Results from these experiments are included in Tables 3, 4 and 5.

## Conclusion and discussion

In this paper we have shown that the Bridge-Prompt framework provides both cutting edge gesture-prediction performance for the standard within-task paradigm as well as strong zero-shot performance on unseen gestures. We believe that this latter case will be essential for any eventual surgical support system. The vocabulary of gestures is too large to learn purely by databases of supervised annotated cases; we should instead plan for situations with weak supervision and novel gestures in deployment. While the Bridge-Prompt framework may not be a component of that eventual system, we believe it makes a significant step towards such a system by demonstrating zero-shot capacity.

## Figures and Tables

**Fig. 2:**
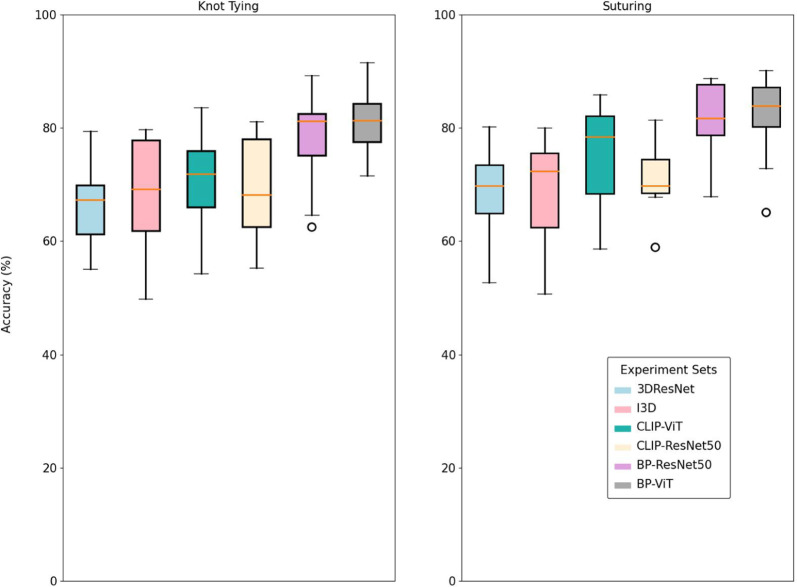
JIGSAWS leave-one-user-out boxplots for main text Table 1

**Fig. 3:**
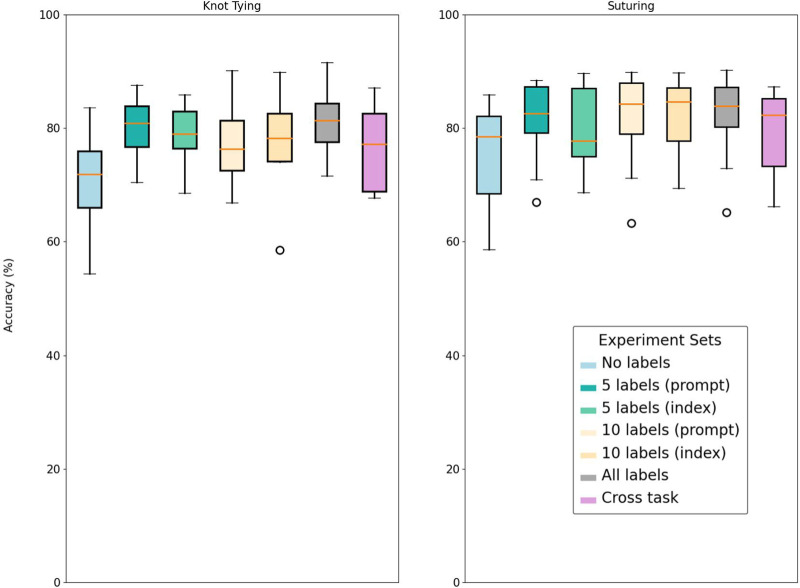
JIGSAWS leave-one-user-out boxplots for main text Table 2

**Fig. 4:**
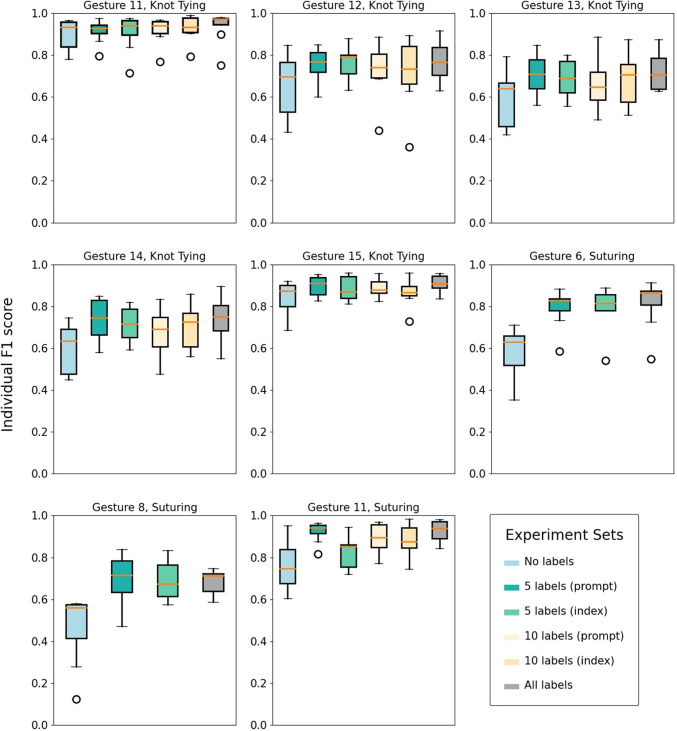
JIGSAWS leave-one-user-out boxplots for main text Table 3

## Appendix

**Table 6:** Gesture reference table in JIGSAWS dataset

Gesture index	Gesture text description
G1	Reaching for needle with right hand
G2	Positioning needle
G3	Pushing needle through tissue
G4	Transferring needle from left to right
G5	Moving to centre with needle in grip
G6	Pulling suture with left hand
G7	Pulling suture with right hand
G8	Orienting needle
G9	Using right hand to help tighten suture
G10	Loosening more suture
G11	Dropping suture at end and moving to end points
G12	Reaching for needle with left hand
G13	Making C loop around right hand
G14	Reaching for needle with right hand
G15	Pulling suture with both hands

See Tables 6, 7 and 8; Figs. 2, 3 and 4.

**Table 7:** Per-gesture performance on RARP-45 dataset (gesture and index)

	G3	G4	G7
No gestures (CLIP)	79.78	72.37	69.47
3 gestures	84.89	**82**.**56**	**77**.**05**
6 gestures	**88**.**85**	–	76.27
All gestures	87.44	78.69	73.38
3 gestures (ind)	85.74	84.38	73.63
6 gestures (ind)	87.74	–	82.17

Best performance referring to specific metric and task

**Table 8:** Comparison of JIGSAWS and RARP-45 dataset

	JIGSAWS	RARP-45
Frame rate (FPS)	30	60
Number of videos	103	36
Average video length (s)	92.1	292.4
Total video length (s)	9488	10,526
Total number of gestures	15	7
Task performed	Suturing; knot tying; needle passing	Radical prostatectomy
Background environment	Lab setting	Operating room
Training time (one validation) (h)	8	10

## Footnotes

1 https://github.com/mx-ethan-rao/Robotic_Gesture_Tools.

2 The original Bridge-Prompt paper uses “Firstly” instead of “First”, and we do not modify it here.

3 Both Bridge-Prompt and 3DResNet methods prescribe the use of weights from another task and training phase before the prompt-tuning phase.

4 Van Amsterdam et al. [6] report results for models that include input data from both video and robotic kinematic streams and thus are not entirely comparable for selecting video encoders. Moreover, the particular architecture, training details and data split cannot be reproduced without access to their codebase. We exclude their RARP-45 reported values as they evaluate on all 45 videos, but only 36 videos in RARP-45 are publicly available.
